# Enhancing the Quality of Surgical Documentation Through the Surgical Tool for Auditing Records (STAR): Experience From a Tertiary Care Hospital in South Asia

**DOI:** 10.7759/cureus.96302

**Published:** 2025-11-07

**Authors:** Omar Mohamed Ozaal Abdul Mubarack, Dileesha Wickramasinghe, Dakshitha P Wickramasinghe

**Affiliations:** 1 General Surgery, University of Colombo, Colombo, LKA; 2 General and Colorectal Surgery, Colchester General Hospital, Colchester, GBR

**Keywords:** clinical audit, low- and middle-income countries, medical records, quality improvement, surgical documentation

## Abstract

Background

Effective record-keeping is crucial for safe surgical care worldwide. While structured tools for auditing records exist, evidence from low- and middle-income countries (LMICs) remains limited. This study was conducted to uphold the standard of surgical record-keeping in a tertiary care center in Sri Lanka using the Surgical Tool for Auditing Records (STAR).

Methods

This prospective, descriptive closed-loop audit included 92 patient records, 44 from Cycle 1 (April 1-14, 2024) and 48 from Cycle 2 (April 15-28, 2024). The evaluation was conducted using the STAR, which assesses documentation based on 50 criteria distributed across six key domains. Structured templates and educational sessions were utilized as interventions. Both audit cycle scores were compared to draw conclusions.

Results

The overall quality of surgical documentation improved significantly, with the mean STAR score rising from 67.34 ± 8.32 to 77.11 ± 7.17 (p < 0.001). Statistically significant improvements were noted across most domains except Consent and Anesthetic Records. Enhancements were observed across all evaluated domains, with the Subsequent Entries category showing the most notable progress (34.8%), while the Anesthetic Records demonstrated the smallest improvement (3.31%). The Discharge Summary section performed exceptionally well, attaining an average score of 94.44 ± 5.61; however, the diagnosis was recorded in only 50% of the reviewed records (n = 24/48). Although the Consent domain improved by 25.89%, it continued to be the lowest-scoring area overall.

Conclusion

Simple, low-cost interventions, such as templates and structured feedback, significantly improved record-keeping quality. Although the STAR has been widely applied globally, this study adds valuable evidence from a Sri Lankan tertiary hospital, offering practical insights for enhancing surgical documentation in LMICs and other resource-limited healthcare systems.

## Introduction

Clinical records, supported by evidence-based medicine, skilled professionals, and emerging digital systems, form the backbone of modern healthcare, serving as both a clinical and administrative tool [1]. They serve as comprehensive documentation of a patient's medical history, current health status, treatment plans, and outcomes [2]. Sound documentation is essential in cases where multiple providers or disciplines are involved, as it ensures that everyone has access to the same information and can coordinate the management seamlessly [2]. Additionally, it also fulfils the legal and regulatory requirements that can be critical in legal situations where proof of care is needed [3]. Comprehensive medical records serve as evidence that a healthcare provider has adhered to the accepted standards of care [3]. The secondary functions of maintaining clinical records are that they serve as sources for research, epidemiology, service planning, resource allocation, and performance monitoring [4].

Therefore, the accuracy and legibility of these documents are paramount to making informed decisions, avoiding medical errors, and maintaining high-quality care [5]. Good clinical record-keeping should reflect complete and timely data gathering, as well as the recording and analysis of clinical information in a way that effectively supports its intended purpose [6].

Despite its importance, clinical record-keeping is often overlooked, leading to poorly maintained notes, illegible or inconsistent entries, offensive comments, and missing information [7]. Poor record-keeping is a common factor cited in malpractice litigation against medical practitioners [7]. There is an urgent need for evidence-based interventions to improve record-keeping standards and ensure that patient information is accurately and comprehensively documented.

A clinical audit enhances the quality of healthcare by assessing clinical practices against established standards to pinpoint areas needing improvement and implement positive changes [8]. Various tools for reviewing clinical records exist, such as the Crawford-Beresford-Lafferty (CRABEL) system, designed for inpatient specialties [9].

The Surgical Tool for Auditing Records (STAR) is a validated system derived from the CRABEL tool. It includes 50 items across six domains assessing surgical documentation quality. STAR has been shown to improve standards of record-keeping [10-12]. Initial Clerking (10 items; 20%), Subsequent Entries (8 items; 16%), Consent (7 items; 14%), Anesthetic Record (7 items; 14%), Operative Record (9 items; 18%), and Discharge Summary (9 items; 18%) were included as domains. The STAR system, known for its high internal consistency (Cronbach’s α = 0.959), provides a structured and reliable alternative to the CRABEL scoring system for assessing record-keeping quality [11]. The total score for individual records is calculated using the following formula: (50 - deducted points) × 2 [11].

Several clinical audits on record-keeping standards have been published across various institutions in Sri Lanka. However, it is unfortunate that no such audits have been published by the National Hospital of Sri Lanka (NHSL) despite it being the leading state hospital in the country. There is a need to raise awareness about the clinical value and research potential of auditing current record-keeping and documentation practices. This study aimed to evaluate and enhance surgical record documentation in a major tertiary care hospital in Sri Lanka using the STAR scoring system within a closed-loop audit cycle. Beyond its local context, this study provides lessons on practical, low-cost strategies for improving documentation that are relevant to other low- and middle-income countries (LMICs), while being potentially adaptable across global healthcare systems.

This study was presented as an abstract at the annual conference of the College of Surgeons of Sri Lanka in 2024.

## Materials and methods

Study design and setting

This investigation was conducted as a prospective, quantitative audit. It focused on surgical documentation from patients admitted to the University Surgical Unit of the NHSL. Ethical approval was obtained from the Ethics Review Committee of the NHSL. All patient identifiers were removed before analysis to maintain confidentiality, and the Committee granted a waiver of individual patient consent. Cycle 1 was performed to establish baseline documentation quality. After interventions, Cycle 2 was completed using the same methodology to assess post-intervention improvement in surgical documentation.

Sample size

Due to logistical constraints and the high patient turnover in the surgical unit, a representative sample of 92 records (44 pre-intervention, 48 post-intervention) was reviewed using systematic random sampling. This approach ensured feasibility while maintaining proportional representation of the full ward record pool (approximately 200 records/month). These records were evaluated to determine the standard and completeness of surgical documentation. And the minimum requirement of STAR was 20 records, which was exceeded.

Study period

Data collection for each audit cycle was carried out over two weeks. The first cycle was conducted from April 1 to April 14, 2024, and the second cycle from April 15 to April 28, 2024. Both cycles included weekend and emergency admissions in accordance with the hospital duty roster to ensure representative sampling.

Data collection

Inpatient case notes from the surgical unit were reviewed manually and scored individually using the STAR. A minimum of 20 patient files is required for valid use of the STAR system. Data collection was performed twice daily at fixed time intervals to minimize temporal bias and ensure consistency in assessing documentation completeness. The project was jointly developed and implemented through collaboration between the NHSL University Surgical Unit and the Faculty of Medicine, University of Colombo.

Interventions

Between the two audit cycles, several strategies were implemented to enhance the quality of surgical documentation. Standardized templates were introduced for operative notes, anesthesia records, postoperative progress, and daily review entries. These templates, designed in alignment with institutional protocols, were distributed among medical officers in the University Surgical Unit. Feedback sessions were organized to present the findings from the first audit cycle. Junior doctors and registrars received targeted training through small-group discussions, emphasizing accurate and comprehensive documentation. They were encouraged to routinely use the standardized templates for operative and postoperative records and to ensure consistent completion and filing within patient case notes.

Statistical analysis

Data were analyzed using IBM SPSS Statistics for Windows, Version 23.0 (Released 2015; IBM Corp., Armonk, NY, USA), the licensed version available at the institution during the study. Descriptive statistics were used to summarize domain-specific and total STAR scores as means ± standard deviations. The distribution of paired differences between Cycle 1 and Cycle 2 was assessed using the Shapiro-Wilk test. When the differences followed a normal distribution, comparisons were made using the paired t-test; otherwise, the Wilcoxon signed-rank test was applied. Statistical significance was set at p < 0.05. Categorical variables were compared using chi-squared or Fisher’s exact tests where appropriate. All analyses followed the ethical principles of the Declaration of Helsinki (1996). The methodology was adapted from previously published STAR-based audits of surgical documentation [13,14].

## Results

**Figure 1:**
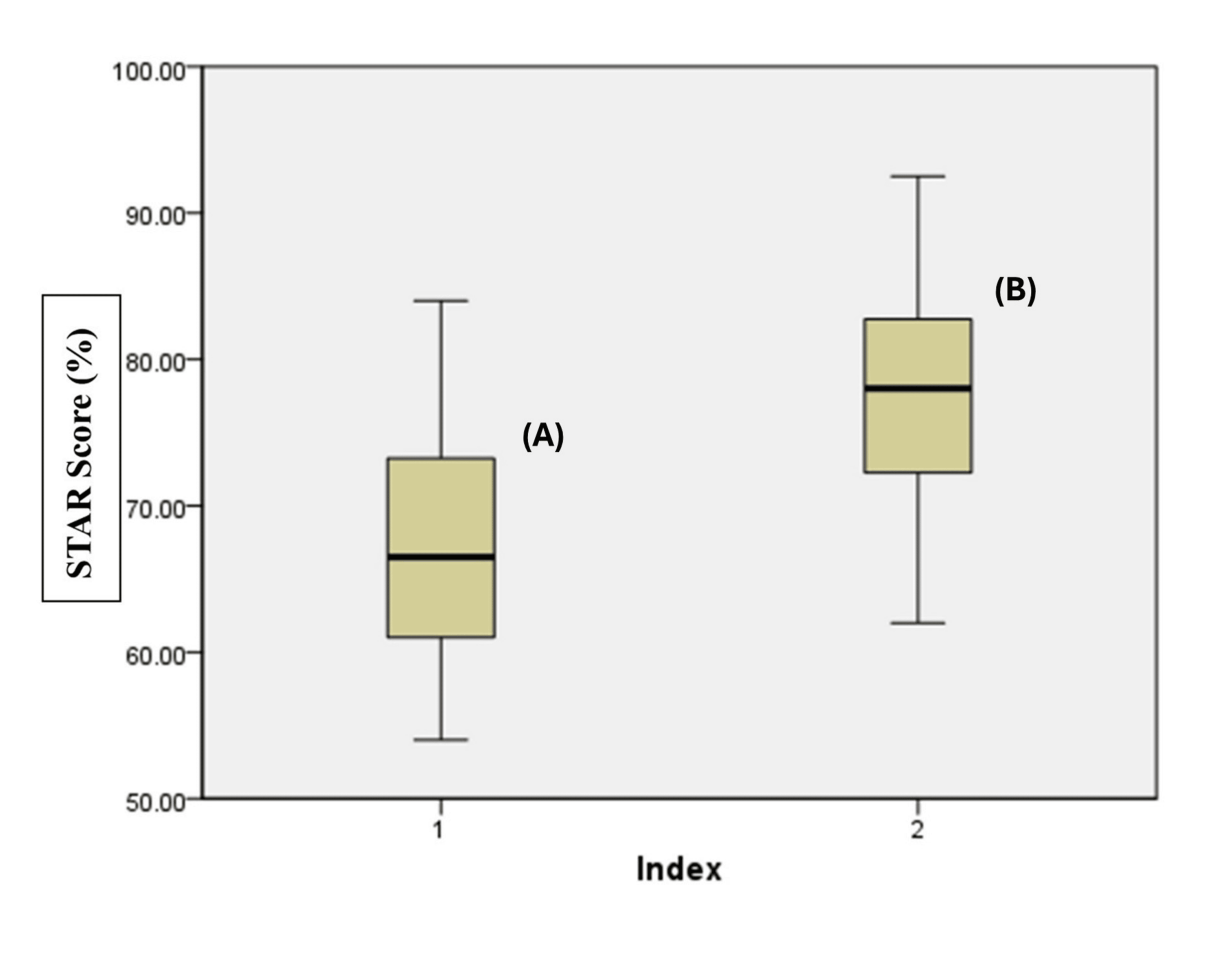
Boxplot comparison of documentation (STAR) scores between Cycle 1 (A) and Cycle 2 (B) (A) Median = 66.2%, IQR = 12.7 (60.8-73.5); minimum = 53.8%; maximum = 84.5%. (B) Median = 78.1%, IQR = 11.9 (71.3-83.2), minimum = 62.4%; maximum = 92.0%. The box represents the interquartile range (IQR), extending from the 25th percentile (Q1) to the 75th percentile (Q3). The horizontal line inside the box indicates the median; the whiskers denote the minimum and maximum values within 1.5 × IQR. STAR: Surgical Tool for Auditing Records.

From April 1 to 28, 2024, we evaluated 92 patient records using the STAR. The results were encouraging, with an overall improvement in the score from an average of 67.34 in Cycle 1 to 77.11 in Cycle 2, as depicted in Figure 1.

**Figure 2:**
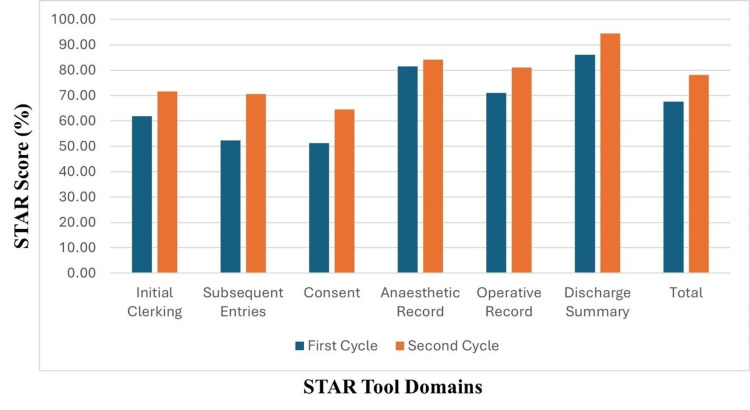
Domain-specific STAR scores for Cycles 1 and 2 STAR- Surgical Tool for Auditing Records.

**Figure 3:**
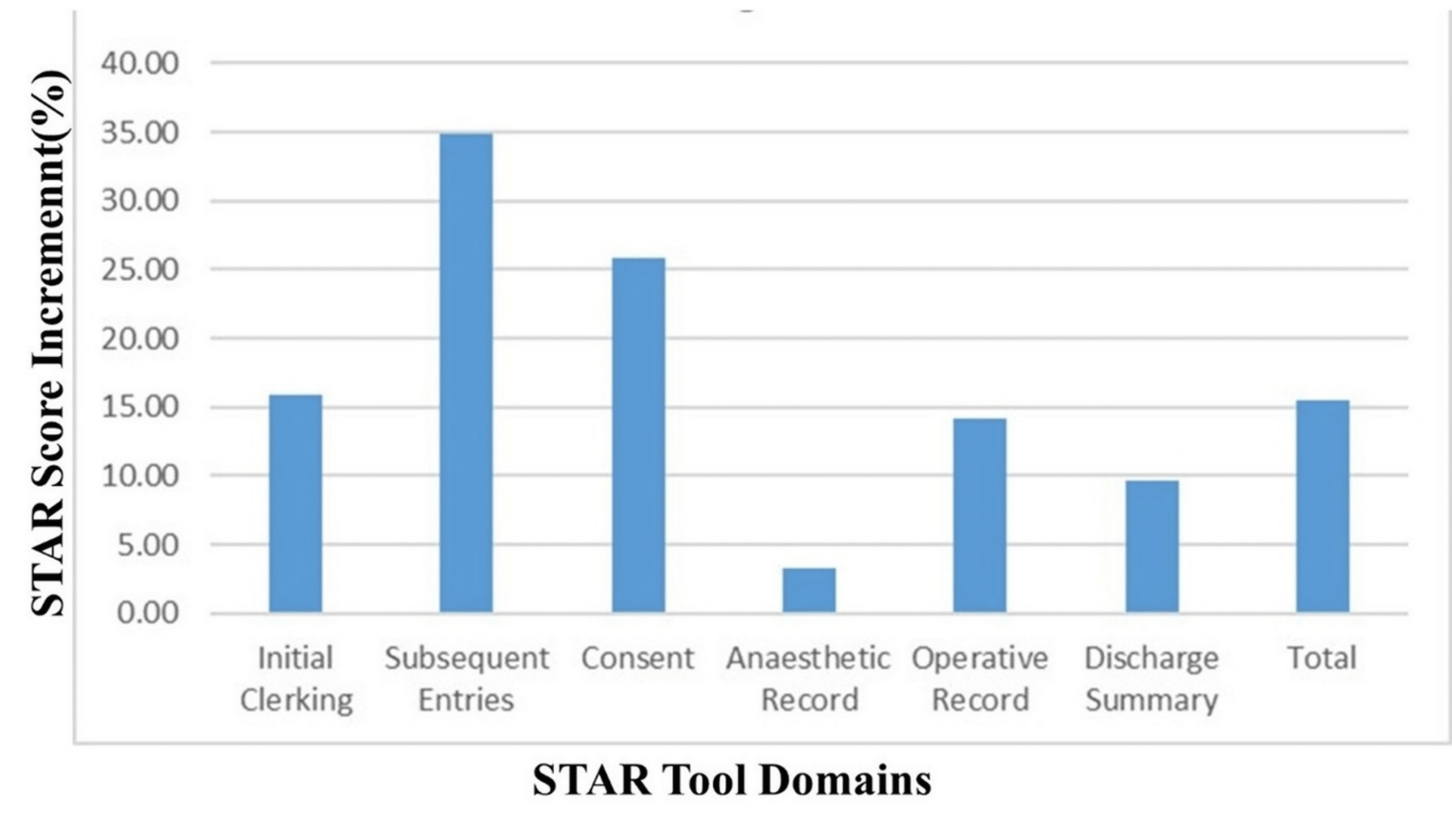
Percentage increase in STAR scores from Cycles 1 and 2 STAR- Surgical Tool for Auditing Records.

Notably, all domains of STAR showed varying levels of improvement from the first cycle to the second, except the Consent and Anesthetic Record domains. The Subsequent Entries domain demonstrated the most significant enhancement (34.8%), followed by Consent (25.89%), while the Anesthetic Record domain showed the smallest improvement (3.31%) (Table 1, Figure 2, Figure 3).

**Table 1:** Domain-wise comparison of STAR scores between audit cycles Mean ± SD STAR scores for each documentation domain during Cycle 1 (N = 44) and Cycle 2 (N = 48) are presented with the percentage increase after intervention. Improvements reflect the effect of structured templates and feedback implemented between cycles. STAR: Surgical Tool for Auditing Records.

Domain	Cycle 1 (mean ± SD)	Cycle 2 (mean ± SD)	Test used	p-value	Statistical significance
Initial Clerking	61.82 ± 13.17	71.14 ± 17.15	Paired t-test	0.0060	Yes
Subsequent Entries	52.34 ± 8.82	71.02 ± 10.64	Paired t-test	<0.0001	Yes
Consent	51.30 ± 25.84	61.69 ± 24.99	Paired t-test	0.0779	No
Anesthetic Record	81.49 ± 9.54	84.09 ± 11.20	Wilcoxon signed-rank	0.0970	No
Operative Record	70.96 ± 16.81	80.81 ± 15.22	Wilcoxon signed-rank	0.0013	Yes
Discharge Summary	86.11 ± 13.15	93.94 ± 5.60	Wilcoxon signed-rank	0.0075	Yes
Total STAR score	67.34 ± 8.32	77.11 ± 7.17	Paired t-test	3.56 × 10⁻⁶	Yes

While none of the domains reached a perfect score of 100 in the second cycle, the Discharge Summary came close with a near-perfect score of 93.94. This is a testament to the overall quality of the records. At the subscale level, significant improvements were observed in several items, including documentation of consultant identity, postoperative signatures, and consent-related risks/complications (exact Fisher's/chi-square p-values reported in Table 2). Other subscales showed numerical but non-significant changes.

**Table 2:** Comparison of STAR subscale compliance between the audit cycles Differences were analyzed using Fisher’s exact or Chi-square tests as appropriate. Exact two-sided p-values are reported; p < 0.05 was considered statistically significant.

Domain	Subscale	Cycle 1, n/N (%)	Cycle 2, n/N (%)	Test	p-value	Statistical significance
Initial Clerking	Patient name	43/44 (97.73%)	48/48 (100.00%)	Fisher's	0.4783	No
	Hospital number	42/44 (95.45%)	48/48 (100.00%)	Fisher's	0.226	No
	Referral source	22/44 (50.00%)	33/48 (68.75%)	Chi-square	0.1054	No
	Consultant	7/44 (15.91%)	22/48 (45.83%)	Chi-square	0.0042	Yes
	Date/time	1/44 (2.27%)	12/48 (25.00%)	Fisher's	0.0019	Yes
	Working diagnosis	15/44 (34.09%)	13/48 (27.08%)	Chi-square	0.615	No
	Investigations/results	37/44 (84.09%)	47/48 (97.92%)	Fisher's	0.0257	Yes
	Management plan	26/44 (59.09%)	31/48 (64.58%)	Chi-square	0.7436	No
	Allergies	44/44 (100.00%)	46/48 (95.83%)	Fisher's	0.4955	No
	Name/post	35/44 (79.55%)	44/48 (91.67%)	Fisher's	0.1352	No
Subsequent Entries	Preop patient name/number	43/44 (97.73%)	48/48 (100.00%)	Fisher's	0.4783	No
	Preop date/time	19/44 (43.18%)	22/48 (45.83%)	Chi-square	0.9636	No
	Preop heading	37/44 (84.09%)	42/48 (87.50%)	Chi-square	0.8655	No
	Preop Pt state/examination	19/44 (43.18%)	28/48 (58.33%)	Chi-square	0.2137	No
	Preop pertinent results	27/44 (61.36%)	47/48 (97.92%)	Fisher's	0.056	No
	Preop plan	39/44 (88.64%)	47/48 (97.92%)	Fisher's	0.1002	No
	Preop signature/name/post	3/44 (6.82%)	9/48 (18.75%)	Fisher's	0.1238	No
	Preop legibility	43/44 (97.73%)	48/48 (100.00%)	Fisher's	0.4783	No
	Postop Pt name/number	39/44 (88.64%)	48/48 (100.00%)	Fisher's	0.0221	No
	Postop date/time	13/44 (29.55%)	33/48 (68.75%)	Chi-square	0.0004	Yes
	Postop heading	36/44 (81.82%)	45/48 (93.75%)	Fisher's	0.1096	No
	Postop Pt state/examination	29/44 (65.91%)	44/48 (91.67%)	Fisher's	0.0038	Yes
	Postop pertinent results	39/44 (88.64%)	47/48 (97.92%)	Fisher's	0.1002	No
	Postop plan	38/44 (86.36%)	47/48 (97.92%)	Fisher's	0.0515	No
	Postop signature/name/post	0/44 (0.00%)	12/48 (25.00%)	Fisher's	0.0003	Yes
	Postop legibility	43/44 (97.73%)	48/48 (100.00%)	Fisher's	0.4783	No
Consent	Patient name/number/date	39/44 (88.64%)	47/48 (97.92%)	Fisher's	0.1002	No
	Operation	19/44 (43.18%)	32/48 (66.67%)	Chi-square	0.04	Yes
	Side and site	20/44 (45.45%)	22/48 (45.83%)	Chi-square	1.0	No
	Benefits	12/44 (27.27%)	18/48 (37.50%)	Chi-square	0.4107	No
	Risks/complications	12/44 (27.27%)	27/48 (56.25%)	Chi-square	0.0094	Yes
	Signatures	33/44 (75.00%)	45/48 (93.75%)	Fisher's	0.0186	Yes
	Name/post	23/44 (52.27%)	26/48 (54.17%)	Chi-square	1.0	No
Anesthetic Record	Name of anesthetist	41/44 (93.18%)	48/48 (100.00%)	Fisher's	0.1055	No
	Preop assessment	44/44 (100.00%)	47/48 (97.92%)	Fisher's	1.0	No
	Drugs and doses	36/44 (81.82%)	46/48 (95.83%)	Fisher's	0.0439	Yes
	Monitoring	44/44 (100.00%)	47/48 (97.92%)	Fisher's	1.0	No
	IVF given	43/44 (97.73%)	47/48 (97.92%)	Fisher's	1.0	No
	Postanesthetic information	40/44 (90.91%)	47/48 (97.92%)	Fisher's	0.1894	No
	Name/signature	3/44 (6.82%)	1/48 (2.08%)	Fisher's	0.3457	No
Operative Record	Name/number/date	41/44 (93.18%)	47/48 (97.92%)	Fisher's	0.3457	No
	Operating surgeon	39/44 (88.64%)	48/48 (100.00%)	Fisher's	0.0221	Yes
	Diagnosis post procedure	8/44 (18.18%)	27/48 (56.25%)	Chi-square	0.0004	Yes
	Description of findings	22/44 (50.00%)	43/48 (89.58%)	Chi-square	0.0001	Yes
	Details of tissues removed	43/44 (97.73%)	48/48 (100.00%)	Fisher's	0.4783	No
	Details of sutures used	30/44 (68.18%)	35/48 (72.92%)	Chi-square	0.7879	No
	Prosthetics with numbers	40/44 (90.91%)	43/48 (89.58%)	Fisher's	1.0	No
	Post-op instructions	44/44 (100.00%)	48/48 (100.00%)	Fisher's	1.0	No
	Surgeon/signature	14/44 (31.82%)	11/48 (22.92%)	Chi-square	0.469	No
Discharge Summary	Name/number/address	44/44 (100.00%)	48/48 (100.00%)	Fisher's	1.0	No
	Admission and discharge dates	44/44 (100.00%)	48/48 (100.00%)	Fisher's	1.0	No
	Discharging consultants	41/44 (93.18%)	48/48 (100.00%)	Fisher's	0.1055	No
	Diagnosis	13/44 (29.55%)	24/48 (50.00%)	Chi-square	0.0741	No
	Pertinent investigations	40/44 (90.91%)	48/48 (100.00%)	Fisher's	0.0486	Yes
	Operations/procedures	43/44 (97.73%)	48/48 (100.00%)	Fisher's	0.4783	No
	Complications	36/44 (81.82%)	48/48 (100.00%)	Fisher's	0.0019	Yes
	Medication on discharge	39/44 (88.64%)	48/48 (100.00%)	Fisher's	0.0221	Yes
	Follow-up	41/44 (93.18%)	48/48 (100.00%)	Fisher's	0.1055	No

Of the subscales of the Discharge Summary, none showed any defects in the second cycle except for stating the diagnosis, which was only available in 50% of the records (n = 24). We believe that the junior doctors are reluctant to make any mistakes when providing a diagnosis. This may be due to a lack of support, experience, and the unavailability of an electronic coding system in the country. We believe this may be the same in most LMICs. But adequate interventions, as shown in our study, will bridge this gap over a long-term period if these audits are repeated at timely intervals.

Despite the Consent domain having the lowest score in both cycles, it showed a significant improvement of 25.89% from the first cycle to the second. This improvement is a promising sign for further enhancement. The benefits of the Procedure subscale were the least scored in the second cycle, found in only 18 (37.50%) of the records. Other subscales with notable deficiencies include the side and site of the procedure and the name and post of the doctor, appearing in only 22 (45.83%) and 26 (54.17%) of the records, respectively (Table 2).

The Subsequent Entries domain, which showed the most significant improvement of 34.8%, is a key area of progress. Its subscale, the postoperative name/signature of the doctor, scored the lowest overall in the first cycle, with zero entries. However, there was a notable improvement in the second cycle, with 12 records (25.0%) including this subscale.

The Anesthetic Record showed the slightest increase in mean score from the first cycle to the second, primarily due to the pre-audit mean score of 81.49. The name of the anesthetist was seen in 100% (n = 48) of the records in the second cycle. However, the name/signature subscale was only shown to be present in one (2.08%) record in the second cycle as opposed to three (6.82%) records in the first, indicating the need for further improvement in this area.

The total STAR score increased significantly from 67.34 ± 8.32 in Cycle 1 to 77.11 ± 7.17 in Cycle 2 (paired t-test, t = -5.317, p = 3.56 × 10⁻⁶). Significant improvements were also observed in Initial Clerking (61.82 ± 13.17 → 71.14 ± 17.15; t = -2.889, p = 0.0060), Subsequent Entries (52.34 ± 8.82 → 71.02 ± 10.64; t = −7.509, p < 0.0001), Operative Record (70.96 ± 16.81 → 80.81 ± 15.22; Wilcoxon W = 172.5, p = 0.0013), and Discharge Summary (86.11 ±13.15 → 93.94±5.60; Wilcoxon W = 87.5, p = 0.0075). Improvements in Consent (51.30 ± 25.84 → 61.69 ± 24.99; t = -1.806, p = 0.0779) and Anesthetic Record (81.49 ± 9.54 → 84.09 ± 11.20; Wilcoxon W = 26.5, p = 0.0970) did not reach statistical significance.

## Discussion

This study was conducted to uphold the standard of surgical record-keeping and aimed to improve adherence to the STAR checklist in the professorial surgical unit services in the NHSL. 

Our results indicate a significant overall improvement in the total STAR score from an average of 67.34 in Cycle 1 to 77.11 in Cycle 2. There were also improvements in documentation quality across all domains following the implementation of several targeted interventions between the two audit cycles. The improvement is a positive indicator of the efficacy of the interventions employed.

The increase in the total STAR score of our study is consistent with the existing literature. Tuffaha et al. reported an increase in the total STAR score from 83.4 to 97.6 [11]. Basu et al. documented an increment in the STAR score from 87 in the first cycle to 93 in the next cycle [15]. Chalikonda et al. also noted a rise from 76.7 to 81 for 10 years [10]. Another descriptive study rather than a full cycle audit by Kakada et al. revealed 98% compliance of their surgical records with STAR [16]. The relative improvement in documentation in our study was more significant compared to the above audits, which is likely due to the lower baseline STAR score of 67.34 ± 8.32. However, the mean score in the second cycle (77.11 ± 7.17) was comparatively lower than those reported in the literature. This discrepancy could be attributed to the shorter study period and limited sample size.

The most notable improvement was observed in the Subsequent Entries domain, which saw a substantial increase of 34.8%. This significant enhancement underscores the importance of regular updates and follow-ups in patient records. The improvement can be attributed to the implementation of structured templates and increased awareness among junior doctors and registrars about the importance of thorough documentation.

The Consent domain remained the lowest-scoring domain in both cycles despite showing an improvement of 25.89%. This indicates that while progress has been made, there are still considerable challenges in ensuring the comprehensive documentation of informed consent. The subscale for "benefits of the procedure" was the least documented, found in only 37.50% (n = 18) of records in the second cycle. Other deficiencies included the documentation of the side and site of the procedure (n = 22, 45.83%) and the name and post of the doctor (n=26, 54.17%). Obtaining informed consent is a fundamental ethical principle in contemporary medical practice and involves an educational process [17]. These findings highlight the need for continuous education and the redesign of consent forms to ensure that all critical information is consistently documented.

During the first cycle, one of the most significant defects was the lack of mentioning the name and post when documenting subsequent entries postoperatively (n = 0). This was also seen in subsequent entries preoperatively (n = 3, 6.82%) and relating to the name of the consultant during the initial clerking (n = 7, 15.91%). This is important since identifying the individuals involved in a patient's care ensures accountability and continuity of care. Another significant issue identified in the first cycle was the omission of the date and time during the initial clerking, which compromises the accuracy and traceability of medical records. Following the interventions, improvements were seen across all subscales mentioned above.

Despite the improvements, the Anesthetic Record domain showed the slightest increase in mean score, with only a 3.31% improvement. The pre-audit mean score for this domain was already high at 81.49, indicating that the baseline quality of anesthetic documentation was relatively good. However, there was the persistence of issues such as the documentation of the name/signature of the anesthetist, which was only present in 2.08% (n = 1) of records in the second cycle, compared to the 6.82% (n = 3) in the first cycle. This suggests the need for targeted interventions in this area to confirm the accuracy and completeness of the information and ensure accountability.

The Operative Record and Discharge Summary domains also showed significant improvements (p = 0.0013 and p = 0.0075, respectively). The use of standardized templates for operative and discharge summaries likely contributed to these enhancements. The near-perfect score of 93.94 ± 5.6 in the Discharge Summary domain in the second cycle is particularly encouraging, suggesting that the interventions were highly influential in ensuring comprehensive discharge documentation.

Our findings resonate with international audits that used the STAR tool in high-income settings, demonstrating that structured interventions can yield meaningful improvements. Importantly, the challenges observed, such as incomplete consent documentation and inconsistent subsequent entries, are not unique to Sri Lanka but are shared across many LMICs. By showing that low-resource, practical measures such as templates and focused education can drive measurable improvements, this study contributes globally relevant evidence that can inform quality improvement initiatives in diverse healthcare systems.

Interventions may continue to focus on areas showing the least improvement, such as the Consent domain. The introduction of a standardized checklist could help ensure that essential information, including the name and post of medical personnel, as well as the date and time, is consistently documented. Regular feedback and recognition of good documentation practices may further motivate ward staff and reinforce adherence to high standards of record-keeping.

Templates, including structured forms and checklists, may support more organized patient management and facilitate high-quality care delivery [18]. They can improve record-keeping by providing a consistent framework for documentation and by reducing the risk of missing critical information. Moreover, they can help systematically aggregate data to evaluate institutional performance and identify areas needing enhancement. The adoption of electronic medical record systems could represent a valuable next step, as previous studies have demonstrated their benefits in improving documentation quality, efficiency, and cost-effectiveness [19].

Expanding the sample size and extending the study period in future audits may provide more comprehensive data and allow stronger comparisons with the existing literature. The findings from this audit may inform similar initiatives across Sri Lanka and other LMICs, contributing to broader efforts to improve the quality and safety of surgical documentation.

Limitations

This study was conducted in a single surgical unit with a relatively small sample size and short study period, which may limit the generalizability of the findings. However, the closed-loop design demonstrates a replicable model for record-keeping audits. The interventions, testing templates, structured education, and feedback are low-cost, scalable strategies that could be readily applied in similar units worldwide. Future multicenter and longer-term studies will help validate and expand these findings. The audit cycles were conducted within a single month, which was split in half, and temporal variation may have influenced results. Future audits should extend data collection over multiple months to minimize time-related bias.

## Conclusions

This closed-loop audit has demonstrated that targeted interventions, including structured templates, feedback, and educational initiatives, significantly improved surgical record-keeping quality in a tertiary hospital in Sri Lanka. While limited to a single unit, the study highlights challenges in documentation that are widely encountered across surgical services, particularly in LMICs. The use of the STAR tool provides a simple yet effective framework for identifying deficiencies and tracking progress. These findings suggest that low-cost, scalable interventions can strengthen documentation practices and patient safety in diverse healthcare contexts, offering lessons that extend well beyond the local setting.

## Appendices

Key messages

What Is Already Known

Surgical documentation is often incomplete, and while tools like the STAR scoring system exist, most published audits come from high-income countries.

What This Study Adds

A closed-loop audit in Sri Lanka shows that simple, low-cost interventions (templates, structured feedback, education) can significantly improve surgical record-keeping.

Why It Matters Internationally

The findings offer a scalable, practical model for improving documentation and patient safety in LMICs, with lessons relevant to healthcare systems worldwide.
